# “*It is today that counts*,* and today everything is fine”*: coping strategies utilized by parents of children treated for cancer who seek psychological support - a qualitative study

**DOI:** 10.1186/s40359-025-02860-4

**Published:** 2025-05-27

**Authors:** Johan Lundgren, Christina Reuther, Paul Farrand, Nina Lutvica, Ella Thiblin, Louise von Essen, Joanne Woodford

**Affiliations:** 1 Department of Women’s and Children’s Health, CIRCLE – Complex Intervention Research in Health and Care, Uppsala University, Dag Hammarskjölds väg 14B, Uppsala, 751 05 Sweden; 2 Division of Nursing Sciences and Reproductive Health, Department of Health, Medicine and Caring Sciences, Linköping University, Norrköping, Sweden; 3 Clinical Education, Development, and Research (CEDAR), Psychology, Faculty of Health and Life Sciences, University of Exeter, Exeter, UK

**Keywords:** Cancer, Childhood cancer, Coping strategies, Cognitive behavioral therapy, Internet-administered intervention, Qualitative research

## Abstract

**Background:**

Childhood cancer treatment completion is a period of vulnerability for parents and is associated with depression, anxiety, restrictions on daily life, and negative socioeconomic consequences. Understanding what helpful and unhelpful coping strategies parents utilize to manage cancer-related distress and concerns may inform the development of tailored psychological support. However, coping strategies used by parents who seek psychological support related to their child’s cancer are not well described. To address this gap, we conducted an embedded semi-structured interview study with parents enrolled into the feasibility study ENGAGE. The overall aim of ENGAGE was to examine the acceptability and feasibility of an internet-administered, guided, low intensity cognitive behavioral therapy based self-help intervention, EJDeR. Study aims were to: (1) describe coping strategies used by parents who seek psychological support after end of treatment to cope with cancer-related distress and concerns and (2) consider these coping strategies to inform ongoing adaptations to the EJDeR intervention, taking potential gender differences in coping and subsequent support needs into consideration.

**Method:**

Seventy-three semi-structured interviews were conducted. Data was coded using inductive manifest content analysis and subsequently triangulated with a secondary theory-driven data analysis guided by the control-based model of coping.

**Results:**

Parents used three primary control coping strategies: *utilizing tools and techniques*, *striving for a healthy and balanced lifestyle*, and *seeking support*. Parents used three secondary control coping strategies: *accepting and refocusing*, *adapting to the situation with help from others*, and *distracting temporarily*. Parents used one disengagement-focused coping strategy: *avoiding and distancing*.

**Conclusions:**

Parents adopted both primary and secondary control coping strategies as well as disengagement-focused coping strategies in accordance with the control-based model of coping. Findings supported the choice of low intensity cognitive behavioral therapy techniques used in the EJDeR intervention to target behavioral and experiential avoidance (i.e., disengagement-focused coping). A need to emphasize the importance of seeking social support in future EJDeR adaptations was identified. Understanding coping strategies used by parents of children off treatment who seek psychological support may inform the development of other psychological interventions for the population.

**Trial registration:**

ISRCTN57233429 (10.1186/ISRCTN57233429; registration date 19/04/2018).

**Supplementary Information:**

The online version contains supplementary material available at 10.1186/s40359-025-02860-4.

## Background

A diagnosis of childhood cancer is one of the most disruptive challenges a parent can experience and is a significant stressor for families [1]. Treatment advances have resulted in increased survival rates [2]. However, the psychological impact on parents is high, with parents experiencing multiple stressors both during and after treatment completion [3]. While treatment completion is an important milestone, it is also a period of vulnerability for the child as well as the parents [4, 5], characterized by fatigue, fear of cancer recurrence/illness uncertainty, and loneliness [5, 6]. Stressors faced during this period include the late effects of childhood cancer and treatment, parenting difficulties, productivity difficulties, and relationship challenges [3, 7]. Research suggests both fathers and mothers have a higher rate of hospital contacts for mental health disorders in the first year after diagnosis up to seven years after diagnosis compared to matched comparisons [8]. Parents of children off treatment (i.e., when the child has completed treatment and at the time is considered successful by a responsible pediatric oncologist) [9] report symptoms of depression (14%) and anxiety (20%) [10, 11], restrictions on daily life [9], and socioeconomic impacts [12, 13].

These findings suggest a need for psychological support among parents of children off treatment, and a subgroup (≈ 20%) of parents residing in Sweden report a need for psychological support from three months to five years after end of treatment [14]. However, only a minority of parents reporting a need for psychological support receive professional support [14]. This unmet need causes suffering not only for parents but also for children. For example, a meta-analysis shows that childhood cancer survivors’ distress is correlated with parents’ distress and symptoms of depression, anxiety, and post-traumatic stress (PTSS), with medium to large effect sizes [15]. Taken together, there is a clear need to develop tailored psychological interventions for parents of children off treatment [5, 8, 14].

To inform the development of psychological support for parents, understanding what coping strategies parents use to manage cancer-related distress and concerns may be important and result in better treatment outcomes [16]. Coping can be defined as cognitive and behavioral efforts employed to manage stressful situations [17, 18]. Stress and coping are two of the most researched fields within psychology [18], and two major theoretical approaches to coping have been developed: the transactional theory of coping [19, 20] and the conservation of resources theory [21, 22], see [18] for a comprehensive review. Many frameworks for classifying coping strategies used in stressful situations have also been developed [23]. One model, informed by the model of perceived control [24] is the control-based model of coping with stress [24]. The model posits that levels of actual and perceived controllability of illness-related stress are important for understanding how children and their parents cope with chronic illness [25, 26].

The control-based model of coping distinguishes between three dimensions: (1) primary control coping; (2) secondary control coping; and (3) disengagement coping [26, 27]. Primary control coping strategies, e.g., emotional expression and problem-solving, are active efforts to change the source of stress (i.e., the situation) or emotional reactions to the stressor [26, 27]. Secondary control coping strategies, e.g., acceptance and cognitive restructuring, are efforts to adapt to or fit with the environment or situation caused by the stressor [25, 26]. Disengagement coping represents efforts to distance oneself physically and/or emotionally from the source of stress [26]. The control-based model of coping may be especially suitable for understanding coping in parents of children off treatment given its emphasis on the role of control in long-term stress in the context of illness [25] and the varied degree of controllability of stressors typically faced in the cancer context [28]. The model has been used to understand how parents cope near the time of the child’s cancer diagnosis or recurrence [28], associations between coping and depression and subsequent mother-child communication about cancer after the child’s diagnosis [29], and associations between depression and coping from diagnosis/recurrence to 5 years after diagnosis [30].

Coping strategies that may be considered primary or secondary, such as active problem-solving and seeking support, are associated with few to no depression symptoms in both fathers and mothers near to their child’s cancer diagnosis [28, 31], and in mothers of children off treatment [30]. Alternatively, strategies that could be categorized as disengagement coping, such as denial and distancing, are associated with high levels of depression symptoms [30, 31] and PTSS [32] in parents of children off treatment. Parents of children off treatment have also been found to use strategies that may be considered primary or secondary control coping, such as acceptance [33, 34], information seeking [34–36], making lifestyle changes to prevent cancer recurrence [34], and religious and spiritual coping [34, 37]. Information-seeking has been identified as a coping strategy used to manage uncertainty and reduce anxiety for parents of children both on and off treatment [38]. Disengagement coping strategies, such as avoidance, denial, mental disengagement, and wishful thinking are also used by parents with children on and off treatment [33, 35, 36]. Further, some differences in the coping strategies adopted by fathers and mothers have been identified. For example, mothers of children on treatment have been found to utilize more social support, information-seeking, and religious coping than fathers [39]. While fathers at the time of diagnosis are more likely to engage in avoidance, denial, and wishful thinking than mothers [28]. Fathers of children off treatment are also more likely than mothers to use mental disengagement, denial, and alcohol/drugs [40].

While research has examined coping strategies used by parents of children off treatment [30, 31, 35, 37, 41], a knowledge gap remains regarding the use of helpful coping strategies [4]. Additionally, coping strategies used by parents who seek psychological support related to the child’s cancer are not well described. To address these gaps, we conducted an embedded semi-structured interview study within the single-arm feasibility study ENGAGE [42, 43] to describe the coping strategies used by parents who seek psychological support after end of treatment to cope with cancer-related distress and concerns. Semi-structured interviews were conducted at baseline with parents enrolled into the ENGAGE feasibility trial [42, 43]. ENGAGE aimed to examine the acceptability and feasibility of an internet-administered, guided, low intensity cognitive behavioral therapy (LICBT) based self-help intervention, EJDeR (Swedish acronym for int**E**rnetbaserad s**J**älvhjälpsprogram för föräl**D**rar till barn som avslutat en behandling mot cance**R**) [44]. EJDeR, developed alongside parent research partners [44, 45], targets symptoms of depression and generalized anxiety disorder (GAD). EJDeR consists of four modules: psychoeducation, behavioral activation, worry management, and relapse prevention [42]. Findings from ENGAGE suggest EJDeR is acceptable and feasible; however, the overall adherence rate was 47.9%, slightly lower than progression criteria (≥ 50%). Adherence differed by first LICBT module chosen. 77% of those choosing behavioral activation as their first LICBT module adhered to the intervention, and 50% of those choosing worry management as their first LICBT module adhered to the intervention [43]. Adherence also differed by gender, with 72% and 42% of fathers and 88% and 56% of mothers adhering to behavioral activation and worry management, respectively [43]. Given adherence concerns, especially for fathers, some adaptations to EJDeR are warranted. Understanding coping strategies used by parents who seek psychological support after end of treatment may help inform these adaptations. For example, research suggests interventions supporting secondary coping strategies, such as acceptance and cognitive reappraisal, can help parents manage the uncertainty of the childhood cancer experience, such as worries about the child’s future [29]. Also, assessing parents’ use of helpful and unhelpful coping strategies may help inform the choice of appropriate therapeutic techniques to work with. Therefore, following the Medical Research Council complex interventions framework [46] we also aimed to use findings to inform adaptations to EJDeR.

### Aims and objectives

Study aims were to (1) describe the coping strategies used by parents who seek psychological support after end of treatment to cope with cancer-related distress and concerns and (2) consider these coping strategies to inform ongoing adaptations to EJDeR, taking potential gender differences in coping and subsequent support needs into consideration. Findings from semi-structured interviews at baseline describing parents’ self-reported concerns have been published elsewhere [7].

## Methods

Reporting follows the consolidated criteria for reporting qualitative research (COREQ) [47], see Supplementary File 1.

### Research team and reflexivity

Interviews were conducted by eight licensed psychologists with 3–30 years of clinical experience, both internal (*n* = 2) and external (*n* = 6) to the research team. None had any previous caring relation to the parents at the time of the interviews. ET, one of the research team members, conducted four interviews.

A multidisciplinary research team with diverse professional and personal backgrounds conducted data analysis. Data analysis was conducted by: JL is a registered nurse with a PhD in Nursing Sciences; NL, a research assistant and cognitive behavioral therapy (CBT) therapist with an MSc in Psychology; ET, a PhD student and licensed psychologist with an MSc in Psychology; and CR, a PhD student with an MSc in Public Health. Peer examination (e.g., reviewing category and subcategory descriptions to ensure data is consistent with findings) was undertaken by three research team members experienced in conducting, teaching, and supervising qualitative research: LvE, a professor in Healthcare Sciences, a licensed psychologist, and principal investigator for ENGAGE; PF, a professor in Evidence-Based Psychological Practice; and JW, an assistant professor in Healthcare Sciences. Five research team members were parents at the time of the study. Three research team members had experience of living with a chronic illness themselves, and five had experience of being closely related to someone living with a chronic illness. The group also included researchers with expertise in CBT, meaning they had an assumption that CBT techniques are helpful in managing psychological distress.

Throughout the analysis process, the research team engaged in reflexive discussions, exploring how their professional roles and personal experiences might have shaped their interpretations. This reflexivity was critical to ensuring the credibility and dependability of the findings.

### Theoretical framework

**Fig. 1:**
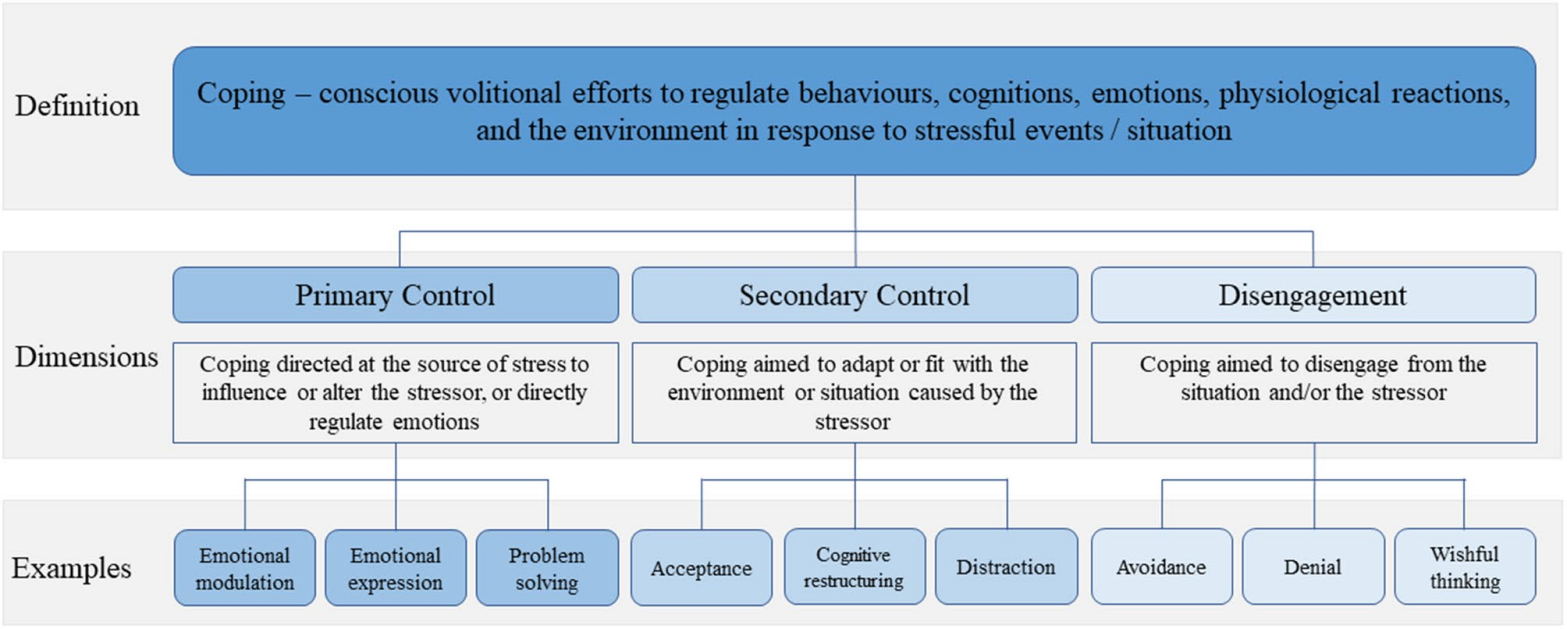
Illustration of control-based model of coping [25]

The underlying research paradigm for the study is pragmatism [48, 49], where methods were chosen to address the two-fold aim of describing coping strategies used by parents who seek psychological support after the end of treatment to cope with cancer-related distress and concerns and to inform ongoing adaptations to EJDeR. In step one, data was coded line-by-line using inductive manifest content analysis [50]. In step two, inductively coded data was triangulated with a secondary theory-driven data analysis guided by the control-based model of coping [25, 51, 52] (Fig. 1).

### Recruitment

Parents were recruited into ENGAGE between July 2020 and November 2020 using two recruitment strategies: (1) postal study invitations and (2) online advertisements. Postal study invitations were sent to parents of children treated for cancer identified via linking the personal identification numbers of children registered on the Swedish Childhood Cancer Registry with parents’ names and addresses on NAVET (a Swedish population registry). Online advertisements were shared via newsletters, social media, and cancer organizations and interest groups’ websites. Inclusion criteria: (1) parent of a child (0–18 years at cancer diagnosis) who completed treatment three months to five years previously (timespan informed by our longitudinal research identifying this as a period of vulnerability for parents [6, 43]); (2) residing in Sweden; (3) able to access the internet, a mobile phone, and BankID (a Swedish digital authentication system); (4) seeking psychological support related to the child’s cancer; and (4) able to read and write Swedish. Exclusion criteria: (1) acute suicidality; (2) a severe and enduring psychological disorder, e.g., bipolar disorder, psychosis, or post-traumatic stress disorder (PTSD) assessed using the Mini-International Neuropsychiatric Interview, M.I.N.I. version 7.0.0 [53]; (3) substance misuse; and (4) currently attending psychotherapy.

Eligibility interviews for study inclusion were completed over the telephone with 76 of the 81 parents who consented to participate in ENGAGE (five parents dropped out of the study before the eligibility interview without providing a reason). Seventy-four parents were invited to and completed the semi-structured interview at baseline (one parent was excluded due to acute suicidality and one parent dropped out). Due to technical failure, one semi-structured interview was not recorded and data is reported for 73 parents (26 fathers, 47 mothers).

### Setting

ENGAGE was coordinated from Uppsala University, Sweden. Eight licensed psychologists conducted the interviews over the telephone. Interviewers were located at Uppsala University, and parents were at a location of their choosing.

### Sample description

Parents had a mean age of 43 years, with 80% born in Sweden. The majority were in a relationship (84%), had completed tertiary level education (71%), were employed (88%), and owned their apartment or house (85%). Children treated for cancer had a mean age of 10 years at the time of the interviews, with the most common diagnosis being leukemia (47%). The mean depression score at baseline (measured with PHQ-9 [54]) was 6.57 (SD = 5.05), and the mean anxiety score (measured with GAD-7 [55]) was 6.06 (SD = 4.74). To facilitate the interpretation of supporting quotations, selected individual parent characteristics and severity of self-reported symptoms of depression and GAD at baseline, are presented in Table 1. Sociodemographic and clinical characteristics for parents and children treated for cancer are presented in Supplementary File 2 and 3, respectively.

**Table 1:** Selected individual parent characteristics

Parent ID	Age (years)	Child’s age (years)	Severity of depression^†^	Severity of anxiety^††^	
Father 1	30–39	5–9	-	-	
Father 2	60–69	0–4	Minimal	Minimal	
Father 3	30–39	5–9	Minimal	Minimal	
Father 4	30–39	5–9	Minimal	Minimal	
Father 5	40–49	5–9	Minimal	Minimal	
Father 6	50–59	5–9	Minimal	Minimal	
Father 7	40–49	10–14	Minimal	Minimal	
Father 8	40–49	10–14	Minimal	Minimal	
Father 9	40–49	10–14	Minimal	Minimal	
Father 10	40–49	10–14	Minimal	Minimal	
Father 11	40–49	10–14	Minimal	Minimal	
Father 12	40–49	15–19	Minimal	Minimal	
Father 13	50–59	15–19	Minimal	Minimal	
Father 14	60–69	20–25	Minimal	Minimal	
Father 15	40–49	5–9	Minimal	Mild	
Father 16	30–39	5–9	Mild	Minimal	
Father 17	40–49	10–14	Mild	Minimal	
Father 18	40–49	10–14	Mild	Minimal	
Father 19	40–49	10–14	Mild	Minimal	
Father 20	30–39	0–4	Mild	Mild	
Father 21	30–39	5–9	Mild	Mild	
Father 22	40–49	5–9	Mild	Mild	
Father 23	50–59	20–25	Mild	Mild	
Father 24	40–49	15–19	Moderate	Mild	
Father 25	40–49	5–9	Moderate	Moderate	
Father 26	50–59	10–14	Moderately severe	Moderate	
Mother 1	30–39	5–9	-	-	
Mother 2	30–39	5–9	Minimal	Minimal	
Mother 3	30–39	5–9	Minimal	Minimal	
Mother 4	30–39	5–9	Minimal	Minimal	
Mother 5	30–39	5–9	Minimal	Minimal	
Mother 6	30–39	5–9	Minimal	Minimal	
Mother 7	30–39	5–9	Minimal	Minimal	
Mother 8	40–49	10–14	Minimal	Minimal	
Mother 9	40–49	15–19	Minimal	Minimal	
Mother 10	40–49	15–19	Minimal	Minimal	
Mother 11	50–59	20–25	Minimal	Minimal	
Mother 12	30–39	5–9	Minimal	Mild	
Mother 13	30–39	5–9	Minimal	Mild	
Mother 14	30–39	5–9	Minimal	Mild	
Mother 15	40–49	5–9	Minimal	Mild	
Mother 16	30–39	5–9	Mild	Minimal	
Mother 17	40–49	10–14	Mild	Minimal	
Mother 18	50–59	15–19	Mild	Minimal	
Mother 19	30–39	0–4	Mild	Mild	
Mother 20	30–39	0–4	Mild	Mild	
Mother 21	30–39	5–9	Mild	Mild	
Mother 22	30–39	5–9	Mild	Mild	
Mother 23	30–39	5–9	Mild	Mild	
Mother 24	30–39	5–9	Mild	Mild	
Mother 25	40–49	5–9	Mild	Mild	
Mother 26	40–49	5–9	Mild	Mild	
Mother 27	40–49	5–9	Mild	Mild	
Mother 28	40–49	5–9	Mild	Mild	
Mother 29	40–49	15–19	Mild	Mild	
Mother 30	50–59	20–25	Mild	Mild	
Mother 31	40–49	5–9	Mild	Moderate	
Mother 32	40–49	5–9	Mild	Moderate	
Mother 33	40–49	5–9	Mild	Moderate	
Mother 34	40–49	10–14	Mild	Moderate	
Mother 35	40–49	5–9	Mild	Severe	
Mother 36	30–39	5–9	Moderate	Mild	
Mother 37	50–59	20–25	Moderate	Mild	
Mother 38	30–39	5–9	Moderate	Moderate	
Mother 39	30–39	10–14	Moderate	Moderate	
Mother 40	40–49	15–19	Moderate	Moderate	
Mother 41	40–49	15–19	Moderate	Moderate	
Mother 42	40–49	10–14	Moderate	Severe	
Mother 43	40–49	15–19	Moderate	Severe	
Mother 44	40–49	15–19	Moderately severe	Mild	
Mother 45	40–49	5–9	Moderately severe	Moderate	
Mother 46	20–29	0–4	Severe	Severe	
Mother 47	40–49	10–14	Severe	Severe	

^**†**^Depression severity cutoffs: 0–4 = Minimal; 5–9 = Mild; 10–14 = Moderate; 15–19 = Moderately severe; 20–27 = Severe [54]

^**††**^GAD severity cutoffs: 0–4 = Minimal; 5–9 = Mild; 10–14 = Moderate; 15–21 = Severe [55]

### Data collection

Interviews lasted between 17 and 67 min (mean 37.5 min, SD 10.9). They were conducted in Swedish by eight licensed psychologists, two of whom had previous experience in qualitative research interviewing. Interviews followed a semi-structured interview guide, exploring coping strategies used by parents to cope with cancer-related distress and concerns, i.e., problems with their physical and mental health and other concerns, such as their relationship with the child, siblings, family, social network, finances, and the healthcare system (Table 2). Interviewers used open-ended follow-up questions to probe around coping strategies used in the context of their cancer-related distress and concerns.

**Table 2:** Semi-structured interview guide

Topic	Questions
Background	(1) When was your child diagnosed with cancer, what was the type of cancer, and when did the treatment end? (2) What does your family situation look like? Has it changed since your child was diagnosed with cancer, for example in terms of partner, siblings living at home, or new siblings?
Main questions	(1) How are you today? (2) Is there anything regarding your current physical and mental health that you relate to your child having been diagnosed with and treated for cancer? If yes, please tell me more about it. (3) Do you think you have any strategy to manage these problems and if so can you tell me a bit about it?
Follow-up questions	(1) If the relationship with the child, persons in the parent’s environment/social network, work and economy, and healthcare were not mentioned in response to question (2), specific questions about whether the parent experienced concerns in these areas are posed, and follow-up questions regarding whether parents have any strategy to manage these problems are asked.

The sample size was predetermined by the number of parents enrolled in ENGAGE, and data collection was not guided by data saturation. Interviews were audio recorded on an Olympus Digital Voice Recorder WS-853. A professional transcriber transcribed them verbatim in Swedish, with identifying information (e.g., names) removed.

### Data analysis

**Fig. 2:**
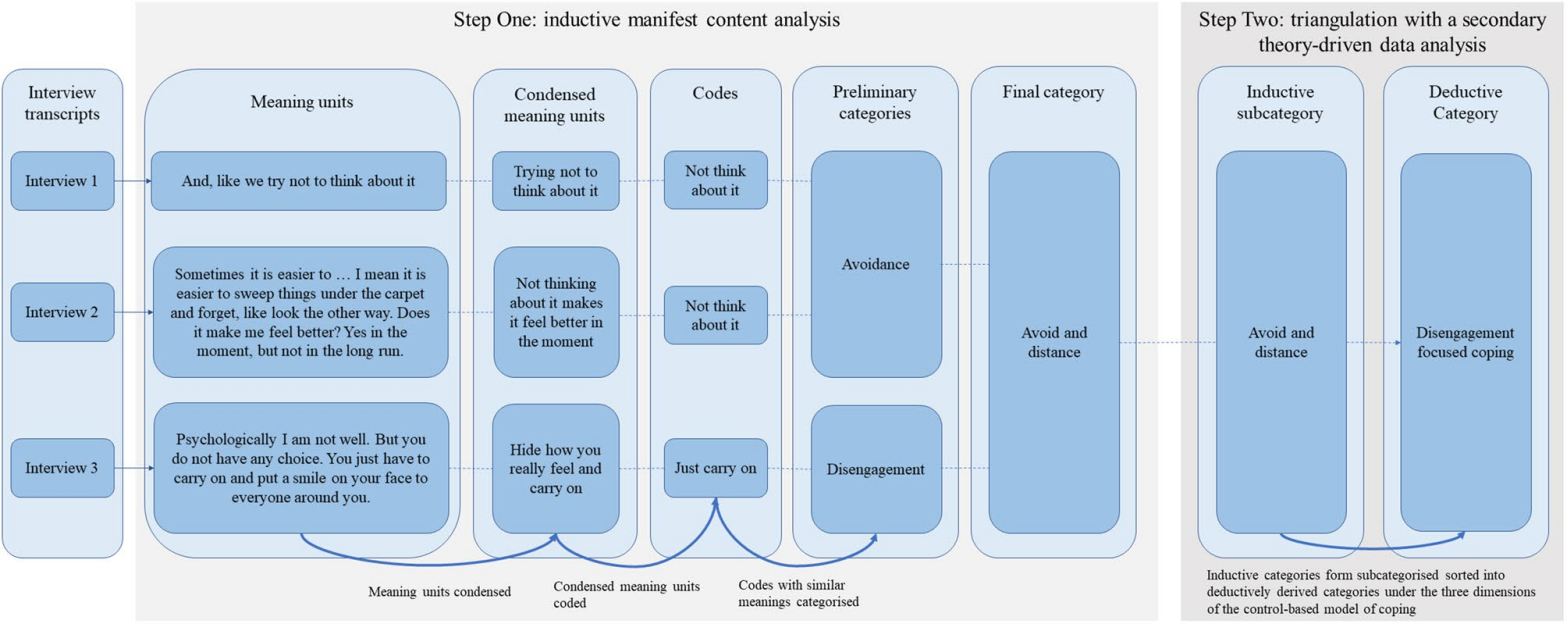
Istration of the two-step data analysis procedure analysis. llu*Note*. The dotted blue and the solid blue arrows indicate the main direction of the analysis, with an increased level of abstraction moving from transcript to finalized categories

NVivo 1.5.1 was used to support data analysis that followed a two-step procedure (Fig. 2).

#### Step one

An inductive manifest content analysis [50] focused on identifying coping strategies used by parents to cope with cancer-related distress and concerns. ET did not analyze the interviews she conducted. Transcripts were read by JL, NL, CR, and ET, who subsequently identified, condensed, and coded meaning units independently. This informed the development of a codebook whereby new code suggestions, coding variations, and unclear codes were discussed at weekly data analysis workshops. When the codebook was revised, interviews were back-checked against the new codebook. At least two authors coded each interview. JL, NL, CR, and ET performed the preliminary categorization of codes into categories with a low degree of interpretation. Following the final iteration of the analysis, decided collaboratively after discussions among JL, NL, CR, and ET, written descriptions of categories were presented to LvE, PF, and JW for peer examination. We aimed to include multiple perspectives in data analysis and interpretation by involving several coders with diverse personal and professional backgrounds. Adopting a multiple-coder approach was considered important to strengthen the dependability of findings and develop a richer and more nuanced understanding of the data [56].

#### Step two

Triangulation with a secondary theory-driven data analysis guided by the control-based model of coping [25, 51, 52] was performed by JL. Inductively derived categories were mapped onto the control-based model of coping, with categories derived from the inductive analysis forming subcategories sorted into deductively derived categories under the three dimensions of the control-based model of coping. Categories were discussed with all co-authors as they were iteratively derived, and the final category structure was developed via peer examination with PF and JW.

### Trustworthiness

To facilitate confirmability, researchers’ pre-understanding was discussed during data analysis workshops. Theoretical triangulation (i.e., a secondary theory-driven data analysis guided by the control-based model of coping [25, 51, 52]); researcher triangulation (i.e., multiple researchers involved in data analysis); and peer examination (i.e., team members not involved in analysis providing feedback on findings) were used to enhance credibility. Disconfirming cases were actively sought.

## Results

Six subcategories representing specific coping strategies were inductively derived during step one of the analysis and subsequently deductively categorized under the three dimensions of the control-based model of coping (Table 3). To aid interpretation, counts relating to how many fathers and mothers respectively mentioned using coping strategies categorised under each inductive subcategory and overall and for each category are reported (Table 3). In total, 77% (*n* = 20) of fathers and 72% (*n* = 34) of mothers mentioned primary coping strategies. 42% (*n*= 11) of fathers and 72% (*n* = 34) of mothers mentioned secondary control coping strategies, whereas 46% (*n* = 12) of fathers and 40% (*n* = 19) of mothers mentioned disengagement-focused coping strategies. As a qualitative study, we did not compare and contrast coping strategies mentioned by fathers and mothers. However, counts are provided to help identify patterns in the data and aid interpretation. Supporting quotations alongside parent ID, relationship to the child (i.e., father or mother) and depression and anxiety severity are provided to strengthen credibility and facilitate transferability of findings.

**Table 3:** Inductive subcategories categorized according to the control-based model of coping, alongside counts for fathers and mothers

Inductive subcategories	Categories		Fathers (*n* = 26)		Mothers (*n* = 47)
		*n*	%	*n*	%
	Primary control coping	**20**	**77**	**34**	**72**
Utilizing tools and techniques		3	12	5	11
Striving for a healthy and balanced lifestyle		13	50	27	57
Seeking support		13	50	22	47
	Secondary control coping	**11**	**42**	**34**	**72**
Accepting and refocusing		9	35	26	55
Adapting to the situation with help from others		1	4	8	17
Distracting temporarily		4	15	14	28
	Disengagement coping	**12**	**46**	**19**	**40**
Avoiding and distancing		12	46	19	40

### Primary control coping

Primary control coping describes parents’ direct efforts to change sources of stress or their emotional reactions to stressors related to their child’s cancer experience. This category includes *utilizing tools and techniques*,* striving for a healthy and balanced lifestyle*,* and seeking support.*

### Utilizing tools and techniques

Parents described adopting a range of tools and techniques to cope with their physical and mental health-related difficulties, as well as broader concerns such as difficulties related to parenting and relationships. Some described using previously acquired problem-solving skills and resources, such as those learnt in psychological therapy:

*“I had*,* was it for two-and-a-half to three years*,* contact with psychiatry. So I feel that I have a lot of tools with me from before*,* and I still know roughly what to do to make everyday life tolerable.”* (Mother 46: severe depression, severe anxiety).

The use of tools and techniques to facilitate effective problem-solving, such as setting realistic goals and breaking down problems to make them more manageable, were also mentioned:

*“Because I’m an engineer*,* I’m problem-oriented*,* so all these problems that we have had*,* I’ve tried to solve them in a practical and engineering-like way.”* (Father 7: minimal depression, minimal anxiety).

Adopting such tools and techniques was perceived by parents as especially important given difficulties with lack of energy and time, and these strategies provided a structured way to manage responsibilities while still attending to their own well-being. Some also described exposing themselves to cancer-related triggers by revisiting reminders and situations related to their child’s experience, helping reduce anxiety and process difficult emotions related to the cancer experience:

*“Sometimes I almost actively try to just*,* well I’ll watch this commercial* [cancer related] *because it’s not that dangerous. ‘What will happen if I actually watch this ad?’ Nothing.*” (Father 17: mild depression, minimal anxiety).

### Striving for a healthy and balanced lifestyle

Striving for a healthy lifestyle was an important coping strategy, particularly for fathers, who mentioned the importance of adapting their child’s diet and that of the wider family. Some fathers described reducing their family’s intake of unhealthy food (e.g., fast food and sugary drinks) and increasing the consumption of healthier, organic food to lower the risk of cancer recurrence, which helped alleviate stress associated with health concerns:

“[Child treated for cancer] *has hardly eaten any sweets at all until this last year…and it’s probably this kind of obsession that I don’t really know if I have any evidence for*,* but there are many studies that show that sugar*,* for example*,* is something that cancer cells like*,* so you feed cancer cells if you consume sugar.”* (Father 6: minimal depression, minimal anxiety).

In addition to dietary changes, striving for a balance in everyday life was voiced as a key strategy to manage stress. Parents emphasized the importance of healthy routines, such as regular physical activity, for improving general well-being, increasing energy levels, and facilitating coping with negative emotions and stress associated with the cancer experience:

*“Regular physical activity is important. It is important to prioritize it* [physical activity] *even when I feel tired and worn out. I have realized that I don’t feel more tired or worn out after a workout*,* but rather the opposite.”* (Father 16: mild depression, minimal anxiety).

Besides physical activity, maintaining everyday routines like household chores and engaging in enjoyable activities such as spending time with family or being in nature were perceived as important to increase energy levels:

*“*[I am] *focusing a bit more on what I want*,* what I like to do and what activities I get more energy from. I have*,* among other things*,* bought a kayak and started to do things that are of interest to me and take time for myself.”* (Father 19: mild depression, minimal anxiety).

Additionally, some parents mentioned using specific strategies to maintain balance in their everyday lives. For example, reducing working hours or prioritizing spending time with family over career advancements to achieve balance in their everyday life:

*“There are some positions that I have been asked to apply for*,* higher management positions than I have today*,* that I have turned down*,* or I have chosen not to apply for because I don’t want to be absent* [from the family] *in the way I know I would be if I got such a position.”* (Father 6: minimal depression, minimal anxiety).

### Seeking support

Parents expressed the importance of actively seeking support from close friends, family members, and colleagues. Talking to others about their feelings and being open with their emotions was described as being helpful in processing the cancer experience:

*“I talk a lot about this* [the childhood cancer experience] *with people that I meet. Some choose not to, but I have always talked about how he *[child treated for cancer] *is and how we are doing and things like that* […] *I feel good about it* [talking]. *It is part of my processing.”* (Father 11: minimal depression, minimal anxiety).

In some cases, this openness led to practical support, such as colleagues and managers adapting parents’ work tasks. Expressing emotions to others also resulted in parents understanding that those close to them shared similar emotions about the cancer experience, which was also perceived as helpful:

*“*You *reflect on how it was and* you *get confirmation that* [other people] *were also sad and scared.”* (Mother 41: moderate depression, moderate anxiety).

Discussions with partners about worry and fears concerning the child’s health further alleviated stress and worry:

*“I also check with his* [the child treated for cancer] *mother what worries she is feeling because she is a little more rational… If I know that* [her] *worry is less than mine*,* I can relax a little. If I know that her worry is the same as mine*,* then I go to the hospital. So that’s the way I handle it.”* (Father 4: minimal depression, minimal anxiety).

Professional support was also identified as helpful for coping with cancer-related concerns and stressors. For instance, some parents had reached out to healthcare providers, counsellors, and community organizations for support:

*“Yes*,* I have sought some help. I attend a course in mental strength that* [name of private company] *has on the web and things like that*,* because I have hit rock bottom myself*,* and I feel that I still have a long way to go to get back up.”* (Father 11: minimal depression, minimal anxiety).

Despite no longer being formally attached to their child’s paediatric cancer care unit at the hospital, parents described maintaining connections with the unit which provided an important sense of security. Seeking therapy, counselling, or religious support was also perceived as helpful in managing mental health difficulties, such as depression and anxiety.

### Secondary control coping

Secondary control coping describes parents’ efforts to adapt to stressors associated with cancer experience by shifting perspectives and managing emotions. This category includes *acceptance and refocusing*, *adapting to the situation with help from others*,* and distracting temporarily*.

### Accepting and refocusing

Some parents described acceptance as an essential way to prevent overwhelming emotions associated with the cancer experience from taking over their daily lives. Acceptance was facilitated by focusing on living day-by-day and recognizing that some difficult periods, such as medical check-ups, were temporary and would pass:

*“It is today that counts*,* and today everything is fine. If something happens tomorrow*,* that is something none of us knows*,* but it is also a very good thing not to* [know]. *It has helped me quite a bit not to think too far into the future.”* (Mother 30: mild depression, mild anxiety).

Accepting that there would be periods when they would feel worse, such as before and after medical check-ups for the child, and recognizing these periods were time-limited, helped parents manage fear and worry. Some parents also voiced that recognizing worrying about cancer recurrence would not affect the actual risk of reoccurrence allowed them to live in the “here and now” and cherish everyday life:

*“Living with that anxiety* [about cancer recurrence] *in the body is not living*,* and something that I still can’t control. It* [cancer recurrence] *will happen regardless of whether I go and worry or not. Instead*,* I tried to turn life around*,* I tried to get back to how it was before and focus on different life projects and the opportunity this journey with* [child] *has given me.”* (Father 19: mild depression, minimal anxiety).

Cherishing the positive things in life and being grateful that their child survived cancer were also described as helpfulpreventing the cancer experience from dominating their life. Parents also expressed gratitude for each day and mentioned how comparing their situation to that of other families facing more severe challenges helped them to appreciate what they had:

*“You have to make the best out of what you have. I mean we’re still better off than many others.”* (Mother 23: mild depression, mild anxiety).

Additionally, parents reminded themselves that experiencing negative emotions did not necessarily mean that something catastrophic was going to happen, helping them to manage their fear of cancer recurrence:

*“I know that* [child treated for cancer] *has*,* for example*,* problems with her stomach after all the treatment and radiotherapy and things she has had done. So I try to look at it as rationally as possible. Yes*,* this* [worry about cancer reccurence] *is a feeling I get. It does not mean that the worst is going to happen.”* (Mother 22: mild depression, mild anxiety).

### Adapting to the situation with help from others

Support from close family and friends helped parents adapt to their new situation. Parents described approaching the cancer experience as a “team” as contributing to them feeling and better able to manage concerns and challenges associated with the cancer experience:

*“No one gets left behind*,* no one is left at home on their own. Everybody is in*,* we will run this* [race together].*”* (Mother 35: mild depression, severe anxiety).

Efforts to adapt to the stressors associated with the cancer experience were facilitated by utilizing help and support from people outside the close family, such as friends who arranged activities for the children, babysitting, and cooking. Utilizing practical support offered by others was described as helpful, resulting in more time to manage everyday life:

*“We learned very early on in the childhood cancer experience*,* to get help damn it*,* with everything. With baking bread and cooking and transport and everything like that. And we did that*,* we were quite good at it*,* we thought* [both] *then and now.”* (Father 11: minimal depression, minimal anxiety).

Parents also described practical support as helpful in managing guilt and feeling that they were not being good enough parents. For example, some parents described how friends had organized pleasurable activities for the siblings of the child treated for cancer, which helped them cope with the cancer experience:

*“I have had great friends that have been there through thick and thin and* [they] *have also been there for our son* [sibling]. *They have taken him out and when there has been a lot on*,* they have taken him with them and arranged things and so on.”* (Mother 9: minimal depression, minimal anxiety).

### Distracting temporarily

Engaging in everyday life activities (e.g., reading a book, running, or working out) or thinking about things other than the cancer experience helped parents to distract themselves from negative emotions. For example, distraction was identified as helpful to stop worry and rumination, enabling engagement with other activities:

*“Because then it* [the worry] *stops when you get out* [of bed] *and think about other things and drink some water.”* (Father 18: mild depression, minimal anxiety).

Some voiced experiencing difficult thoughts around “*what if*” the cancer treatment had failed, and engaging in activities such as cuddling their children, household chores, or work were perceived as helpful in distracting themselves from these thoughts:

*“*[I] *can clean the kitchen counter* […] *It kind of gives a temporary calm.”* (Mother 7: minimal depression, minimal anxiety).

While distraction was experienced as helpful in specific situations or for shorter periods, some parents recognized it as unhelpful in the longer term. For example, some considered distraction as something preventing them from problem-solving their challenges:

*“I mean what I do is to distract myself*,* and it works temporarily. I do other things. I work and I quite simply do other things*,* to distract myself. And it works well*,* but it doesn’t solve the basic problem so to speak. So* [I] *have quite good strategies*,* I think*,* but only how to manage it*,* not how to solve it.”* (Mother 26: mild depression, mild anxiety).

### Disengagement-focused coping

Disengagement-focused coping describes parents’ efforts to avoid sources of stress or their own emotional reactions to stressors related to their child’s cancer experience. This category includes *avoiding and distancing.*

#### Avoiding and distancing

Parents described ways of avoiding or distancing themselves from cancer related stress, such as avoiding thinking or talking about the illness and staying away from specific places or situations that triggered painful memories:

*“When we get a palliative patient* [at work] *I do not attend to that* [patient] *because it becomes too tangible for me.”* (Mother 46: severe depression, severe anxiety).

While this strategy provided short-term relief, parents acknowledged that avoidance was unhelpful in the longer term, often prolonging emotional distress:

*“Sometimes it’s easy for me to sweep things under the carpet and forget and just look the other way.* […] *And it does* [feel better] *in the moment but in the long run it doesn’t.”* (Father 3: minimal depression, minimal anxiety).

There were important nuances in the way avoiding and distancing were described. Some described needing to “*just carry on*” and maintain appearances to others, hiding their feelings to enable themselves to function in everyday life. However, parents recognized this strategy as interfering with opportunities to reflect on the cancer experience and seek help:

*“You just had to keep the days together to make it work.* […] *I have probably never sat down and pondered about what happened and actively tried to get help. Instead*,* it has been like… well I’ve just kind of carried on.”* (Father 21: mild depression, mild anxiety).

Some described distancing themselves from their own emotions to prioritize the needs of their children or others close to them, often feeling as though they had no choice but to continue functioning:

*“You had to put on a mask for* [child treated for cancer’s] *sake and just keep going*,* even though you just wanted to hide in a room and sit and cry and feel sorry for yourself*,* but you can’t do that. I feel that mentally I am not well*,* but you have no choice. You just have to keep going and put a smile on your face for everyone around you and just simply function.”* (Mother 28: mother, mild depression, mild anxiety).

Parents also distanced themselves from the cancer experience by viewing it as a closed chapter in life, describing a desire to “*move on*” and return to normality:

*“I do not know if I have the strength to deal with those parts*,* I just want to put a lid on it and move on.”* (Father 19: mild depression, minimal anxiety).

To facilitate “*moving on*”, they described avoiding cancer-related contexts, such as cancer support organizations:

*“I don’t want to get involved with a cancer support organization and things like this. It was a chapter in life then. I want to remember it* [the cancer experience] *but I don’t want it to go on and on.”* (Father 17: mild depression, minimal anxiety).

## Discussion

This study aimed to describe the coping strategies used by parents of children treated for cancer who seek psychological support after the end of treatment to cope with cancer-related distress and concerns and consider these coping strategies to inform ongoing adaptations to EJDeR, taking potential gender differences in coping and subsequent support needs into consideration. Inductively derived subcategories were mapped onto the control-based model of coping [25]. Parents used a diverse range of primary control coping strategies, including *utilizing tools and techniques*, *striving for a healthy and balanced lifestyle*, and *seeking support*. Parents also used secondary control coping strategies, including *accepting and refocusing*, *adapting to the situation with help from others*, and *distracting temporarily*. Parents used one disengagement-focused coping strategy *avoiding and distancing*. However, this strategy was recognized as unhelpful in the long term, interfering with reflecting on the cancer experience and seeking appropriate support.

Parents utilized various primary control strategies, including problem-solving and setting realistic goals, to direct efforts to change sources of stress or their emotional reactions to stressors related to their child’s cancer experience. This finding aligns with previous research on coping strategies used by parents of children with cancer both on [28–30, 57–59] and off [30, 31, 41] treatment, which indicates that they frequently use problem-focused coping strategies. Parents recognised that these strategies helped them cope with anxiety, low mood and stress more effectively, which is in line with research showing primary control coping to be associated with lower levels of depression and anxiety [60]. Positive problem orientation (i.e., viewing problems as challenges that can be solved) has also been found to be associated with higher mental health-related quality of life and less anxiety in caregivers (primarily parents) of children off treatment [41].

An additional primary control coping strategy used by parents was actively seeking balance in life, e.g., maintaining everyday routines and engaging in physical activity, which parents found helpful in coping with low mood and increasing energy levels. Research suggests that re-engagement in leisure and self-care activities facilitates adaption to a “normal life” after end of treatment [61]. This is of particular importance given engagement in meaningful activities is associated with psychological well-being [62], and physical activity is associated with a lower risk of depression [63] and anxiety [64]. However, our previous research suggests some parents continue to experience restrictions in leisure activity after end of treatment, and those experiencing PTSS report significantly more restrictions compared to parents without PTSS [9]. Additionally, parents of children with cancer commonly fail to meet recommended physical activity guidelines, which may compromise both their own and the health of their child [65]. Supporting parents in using helpful coping strategies such as engaging in leisure and physical activity may positively impact their mental and physical health.

Social support was also highlighted as helpful in processing the cancer experience and alleviating stress. Support from family, friends, and the wider community has been found to promote resilience and post-traumatic growth in parents of children on [59, 66, 67] and off [67] treatment. However, parents also report relationship challenges and a perceived lack of social support [7], leading to isolation from others [68]. Given social support is associated with lower anxiety and PTSS [67], as well as reduced psychological distress [31], supporting parents to seek professional and social support should be a component of interventions aimed at promoting psychological well-being in parents. Furthermore, healthcare providers should consider routinely assessing social support in parents to identify those at risk of isolation and provide targeted resources or referrals to improve social support systems.

Interestingly, information-seeking, a common coping strategy identified as being used by parents of children off treatment in other research [34, 36], was not mentioned. This could be due to the timing of the interviews, which took place several months to years after treatment had ended. Previous research identifying information seeking as a coping strategy included parents of newly diagnosed children [69] or those who only recently ended treatment [34]. While parents in this study appreciated the possibility of contacting the pediatric oncology unit, other studies suggest providing information in the long term is important, with unmet information needs persisting years after end of treatment [36]. Religious or spiritual coping strategies are also used by parents of children on [59] and off treatment [34] and are associated with lower levels of PTSS and symptoms of depression and anxiety [70], but were not mentioned by parents in this study. This finding could be attributed to the predominantly Swedish-born sample, given Sweden is a largely secular society in which religious or spiritual perspectives on life are seldom addressed in healthcare [71]. Importantly, Sweden is becoming a more diverse and multicultural, with 20% of the population being foreign-born [72]. This may pose challenges to a predominantly secularized healthcare context, and future research should explore concerns and coping strategies used by parents from ethnic minority groups to inform appropriate support adapted to their concerns and the potential influence of cultural and religious factors [73].

Lastly, our findings align with research suggesting parents adopt disengagement-focused coping strategies to distance themselves from the cancer experience on and off treatment [28, 60]. The use of disengagemen-focused coping strategies is associated with higher levels of depression and anxiety both on and off treatment [60], and some parents in the present study recognized avoidance as bening unhelpful in the long term, often prolonging emotional distress. Fathers, in particular, were more likely than mothers to mentioned disengagement strategies, which is also consistent with other research [28]. Research suggests men are more likely than women to use avoidance coping strategies, which are associated with less help-seeking behaviors and an increased risk of alcohol and other substance misuse [74–76]. Emotional avoidance is also associated with depression severity and symptoms PTSD [77]. Research also suggests fathers of children off treatment may experience delayed PTSD onset [78] and are more likely to experience long-term financial and occupational challenges, e.g., negative impacts on career and pension development [13], and reduced earnings and increased sick leave, after their child’s treatment or death [79]. In addition, secondary control coping strategies were mentioned more often by mothers than fathers. Secondary control coping is considered helpful in the context of chronic illness and can help parents manage the uncertainty of the childhood cancer experience, such as worries about the child’s future [29]. Targeted interventions to encourage primary and secondary coping strategies, alongside workplace policies to support occupational challenges, may benefit fathers throughout the disease trajectory [13].

### Implications for adaptations to the EJDeR intervention

Overall, results support using behavioral activation and worry management as core LICBT techniques in the EJDeR intervention and point to possible adaptations. Problem-solving was mentioned by parents as a primary control coping strategy. Problem-solving is included in worry management [44], and findings support the use of this therapeutic technique. An additional primary control coping strategy mentioned by parents was actively striving for a healthy and balanced lifestyle, for example, making time for physical activity, spending time with their children, and reducing working hours. This strategy is in line with behavioral activation, which targets behavioral avoidance, i.e., disengagement from pleasurable, routine, and self-care activities [44]. The finding parents use avoidant coping strategies further supports the use of behavioral activation and worry management as techniques designed to reduce behavioral and experiential avoidance, which are maintaining factors in depression and GAD [80, 81].

Results also suggest a need for adaptations. Parents mentioned the importance of seeking support from family, friends, and the wider community. Despite difficulties utilizing social support being common among parents of children off treatment [7, 68], social support is not explicitly acknowledged in EJDeR. Future adaptations will include information to facilitate utilizing social support. The use of avoidant coping strategies is a core feature of PTSD [82]. Avoiding thinking or talking about the cancer experience and external reminders of the experience may contribute to the development and maintenance of PTSS. Techniques targeting PTSS were not included in EJDeR, as there was a lack of evidence supporting the use of internet-administered CBT for PTSS at the time of development [83]. However, the evidence base for internet-administered CBT has grown [84], and material focused on alleviating PTSS will be included in the future adaptation of EJDeR. Fathers mentioned avoidant coping strategies somewhat more than mothers. More emphasis on the role of avoidance in the development and maintenance of symptoms of depression, GAD, and PTSS may increase the perceived relevancy of EJDeR for fathers, resulting in improved adherence rates.

As well as informing adaptations to EJDeR, findings have broader implications for developing psychological interventions for parents. Some psychological interventions for parents of children off treatment include CBT-based techniques to improve coping skills. A computer-mediated group support was found to improve symptoms of depression in mothers and anxiety in fathers [85]. An online group CBT intervention via video-conferencing for parents of children treated for cancer was found to be acceptable and feasible [86]. However, the intervention did not significantly improve health-related quality of life, depression, or anxiety [87]. Given the lack of high-quality evidence, there is an urgent need to develop tailored psychological support to help parents cope with and adapt to the period after end of treatment [88]. Assessing coping strategies may guide the selection of appropriate therapeutic techniques, enhancing the overall effectiveness of interventions. Our findings provide valuable insights into appropriate techniques for the population to facilitate the use of helpful coping strategies and reduce the use of problematic coping strategies. Findings may also be transferable to parents of children with other serious illnesses.

### Study limitations

First, the transferability of findings may be limited given parents were mainly Swedish-speaking mothers with high levels of education, born in Sweden, and parents were not required to meet diagnostic criteria for major depressive disorder or GAD. Given fathers reported using disengagement-focused strategies more often than mothers and secondary-control coping less often than mothers, there may be important gender differences in coping, which could be examined in future research. Further limiting transferability was heterogeneity in the length of time since the child completed cancer treatment, and coping strategies used by parents may change over time. Second, the study may be conceptualized as a large interview study [89], and complexities and nuances within the data may not be captured when managing large amounts of qualitative data [90]. However, as semi-structured interviews did not solely focus on coping strategies (with findings describing parents’ self-reported concerns published elsewhere [7]), a large sample size may be considered necessary to achieve sufficient information power [91]. Interviews varied in length (17–67 min), which may have limited the depth and nuance of responses. This variation could stem from several licensed psychologists conducting the interviews, most lacking qualitative interviewing experience. Third, although we did not aim to explore relationships between primary and secondary control coping, they may be important to consider. The relationship between primary and secondary coping strategies is complex and contextually dependent [18], and different strategies may be adopted at different time points across the cancer disease trajectory. Fourth, the control-based model of coping [25, 51, 52] was not always straightforward to use. While distraction is considered a secondary control coping strategy, prolonged distraction to avoid stressors could be viewed as behavioral avoidance, a disengagement-focused coping strategy [27]. Other conceptualizations of coping, such as classifying coping strategies as problem focused or emotion focused [92], may have been easier to apply but may have also missed important nuances. Finally, using a deductive approach may have resulted in data not fitting the control-based model of coping being overlooked or forced into the model. However, the combination of inductive and deductive analyses suited our pragmatic aim of describing coping strategies used by parents and their potential to inform intervention development. Additionally, triangulation of a theoretical framework with empirical data strengthens the credibility and confirmability of findings. The use of disconfirming case analysis, data analysis workshops, independent coders, and peer review are further strengths of the study, despite the limitations mentioned above.

## Conclusions

This is the first study to describe the coping strategies used by parents who seek psychological support after the end of treatment to cope with cancer-related distress and concerns. Parents adopted various strategies aligned with the control-based model of coping [25, 51, 52]. The findings support using LICBT techniques in EJDeR, targeting behavioral avoidance associated with depression and GAD. Understanding these coping strategies will inform further adaptations to EJDeR. Findings also have broader implications for the development of psychological interventions for parents of children treated for cancer and other chronic illnesses.

## Electronic supplementary material

Below is the link to the electronic supplementary material.

Supplementary Material 1

Supplementary Material 2

Supplementary Material 3

## Data Availability

The datasets generated and/or analyzed during the current study are not publicly available due to privacy or ethical restrictions but are available from the corresponding author on reasonable request.

## Abbreviations

CBT: Cognitive behavioral therapy
GAD: Generalized anxiety disorder
LICBT: Low intensity cognitive behavioral therapy
PTSD: Post-traumatic stress disorder
PTSS: Post-traumatic stress
