# Supplementary material for: “It is today that counts, and today everything is fine”: coping strategies utilized by parents of children treated for cancer who seek psychological support - a qualitative study
Source: BMC Psychol. 2025 May 27;13:565. doi: 10.1186/s40359-025-02860-4 (PMC12108027; doi:10.1186/s40359-025-02860-4)
Supplement: Supplementary file 2 — Supplementary Material 2 [file 40359_2025_2860_MOESM2_ESM.docx]

**Supplementary File 2.** **Sociodemographic and clinical characteristics for fathers (N=26) and mothers (N=47)**

| **Sociodemographic and clinical characteristics** | **Total (N=73)**  **n (%)** | **Fathers (N=26)**  **n (%)** | **Mothers (N=47)**  **n (%)** |
| --- | --- | --- | --- |
| Gender |  |  |  |
| Fathers | 26 (35.6) | 26 (100.0) | - |
| Mothers | 47 (64.4) | - | 47 (100.0) |
| Age (years) |  |  |  |
| Mean (SD) range | 42.7 (7.0) 26-62 | 45.9 (7.1) 36-62 | 41.0 (6.4) 26-59 |
| Number of children |  |  |  |
| Median (range) | 2 (1-5) | 2 (1-4) | 2 (1-5) |
| Age of children^†^ |  |  |  |
| Mean (SD) range | 12.0 (7.2) 0.5-37 | 13.7 (8.5) 2-37 | 11.0 (8.2) 0.5-29 |
| Highest level of education |  |  |  |
| Lower secondary | 1 (1.4) | 1 (3.8) | 0 (0.0) |
| Upper secondary | 15 (20.5) | 6 (23.1) | 9 (19.1) |
| Post-secondary non-tertiary | 3 (4.1) | 0 (0.0) | 3 (6.4) |
| Tertiary | 52 (71.2) | 17 (65.4) | 35 (74.5) |
| PhD | 2 (2.7) | 2 (7.7) | 0 (0.0) |
| Housing situation |  |  |  |
| Rental | 8 (11.0) | 3 (11.5) | 5 (10.6) |
| Apartment ownership | 17 (23.3) | 6 (23.1) | 11 (23.4) |
| House ownership | 45 (61.6) | 16 (61.5) | 29 (61.7) |
| Other | 3 (4) | 1 (3.8) | 2 (4.3) |
| Region of birth |  |  |  |
| Nordic countries^*^ | 61 (83.6) | 21 (69.2) | 40 (85.1) |
| Asia | 6 (8.2) | 2 (7.7) | 4 (8.5) |
| Europe (excl. Nordic countries) | 5 (6.8) | 2 (7.7) | 3 (6.4) |
| Africa | 1 (1.4) | 1 (3.8) | 0 (0.0) |
| Employment status |  |  |  |
| Employed | 64 (87.7) | 24 (92.3) | 40 (85.1) |
| Unemployed | 9 (12.3) | 2 (7.7) | 7 (14.9) |
| Previous psychological treatment |  |  |  |
| Yes | 40 (54.8) | 12 (46.2) | 28 (59.6) |
| No | 33 (45.2) | 14 (53.8) | 19 (40.4) |
| Relationship status |  |  |  |
| Partner | 61 (83.6) | 22 (84.6) | 39 (83.0) |
| Single | 12 (16.4) | 4 (15.4) | 8 (17.0) |
| If partner, cohabiting^‡^ |  |  |  |
| Yes | 60 (98.4) | 21 (95.5) | 39 (100.0) |
| No | 1 (1.6) | 1 (4.5) | 0 (0.0) |
| Physical health problems^§^ |  |  |  |
| Yes | 23 (31.5) | 9 (34.6) | 14 (29.8) |
| No | 50 (68.5) | 17 (65.4) | 33 (70.2) |
| Type of physical health problem^¶^ |  |  |  |
| Diseases of the musculoskeletal system and connective tissue | 9 (12.3) | 4 (15.4) | 5 (10.6) |
| Endocrine, nutritional and metabolic diseases | 5 (6.8) | 1 (3.8) | 4 (8.5) |
| Diseases of the genitourinary system | 3 (4.1) | 1 (3.8) | 2 (4.3) |
| Diseases of the circulatory system | 2 (2.7) | 2 (7.7) | 0 (0.0) |
| Diseases of the digestive system | 2 (2.7) | 1 (3.8) | 1 (2.1) |
| Diseases of the nervous system | 1 (1.4) | 0 (0.0) | 1 (2.1) |
| Diseases of the respiratory system | 2 (2.7) | 0 (0.0) | 2 (4.3) |
| Diseases of the skin and subcutaneous tissue | 1 (1.4) | 0 (0.0) | 1 (2.1) |
| Neoplasm | 1 (1.4) | 1 (3.8) | 0 (0.0) |
| Other cannot classify | 3 (4.1) | 1 (3.8) | 2 (4.3) |
| Previous traumatic/difficult life event^§^ |  |  |  |
| Yes | 59 (80.8) | 19 (73.1) | 40 (85.1) |
| No | 14 (19.2) | 7 (26.9) | 7 (14.9) |
| Type of previous traumatic/difficult life event^¶^ |  |  |  |
| Child’s cancer disease | 34 (46.6) | 14 (53.8) | 20 (42.6) |
| Death in family and miscarriage | 21 (28.8) | 3 (11.5) | 18 (38.3) |
| Severe disease/illness own/family/friends | 17 (23.3) | 2 (7.7) | 15 (31.9) |
| Divorce or separation | 12 (16.4) | 2 (7.7) | 10 (21.3) |
| Exposure to violence or sexual abuse | 6 (8.2) | 1 (3.8) | 5 (10.6) |
| Suicide/suicide attempt among family/friends | 4 (5.5) | 2 (7.7) | 2 (4.3) |
| War/terrorist attacks | 3 (4.1) | 0 (0.0) | 3 (6.4) |
| Other traumatic experiences | 13 (17.8) | 6 (23.1) | 7 (14.9) |
| ^†^Includes all children of parents, not only the child treated for cancer.  ^*^Nordic countries represented in the study sample include Denmark, Finland, Norway, and Sweden.  ^‡^Percentage of cohabiting is based on the parents having a partner.  ^§^More than one physical health problem or previous traumatic/difficult life event may be reported by each parent.  ^¶^Percentage of type of physical health problem or type of previous traumatic/difficult life event is based on N=73. | | | |
