# Supplementary material for: “It is today that counts, and today everything is fine”: coping strategies utilized by parents of children treated for cancer who seek psychological support - a qualitative study
Source: BMC Psychol. 2025 May 27;13:565. doi: 10.1186/s40359-025-02860-4 (PMC12108027; doi:10.1186/s40359-025-02860-4)
Supplement: Supplementary file 3 — Supplementary Material 3 [file 40359_2025_2860_MOESM3_ESM.docx]

**Supplementary File 3.** **Sociodemographic and clinical characteristics for children treated for cancer (N=68)**

|  | **Parent self-report data**  **(N = 68)^†^** | **Fathers (N = 23)** | **Mothers (N = 45)** |
| --- | --- | --- | --- |
| Current age (years) |  |  |  |
| Mean (SD) range | 10.5 (5.1) 2–24 | 11.0 (5.1) 4-24 | 10.3 (5.1) |
| Gender |  |  |  |
| Female | 30 (44.1) | 8 (34.8) | 22 (48.9) |
| Male | 38 (55.9) | 15 (65.2) | 23 (51.1) |
| Cancer diagnosis |  |  |  |
| Blastoma | 11 (16.2) | 4 (17.4) | 7 (15.6) |
| Carcinoma | 1 (1.5) | 0 (0.0) | 1 (2.2) |
| CNS tumor | 8 (11.8) | 1 (4.3) | 7 (15.6) |
| Germ cell tumor | 2 (2.9) | 0 (0.0) | 2 (4.4) |
| Leukemia | 32 (47.1) | 12 (52.2) | 20 (44.4) |
| Lymphoma | 9 (13.2) | 3 (13.0) | 6 (13.3) |
| Sarcoma | 4 (5.9) | 2 (8.7) | 2 (4.4) |
| Do not know | 1 (1.4) | 1 (4.3) | 0 (0.0) |
| Data are number (%) unless stated otherwise.  Percentages may not always total 100 due to rounding.  **^†^**Data for children with both parents participating in the study (n = 5), are only included once. | | | |
